# The impact of service and hearing dogs on health-related quality of life and activity level: a Swedish longitudinal intervention study

**DOI:** 10.1186/s12913-018-3014-0

**Published:** 2018-06-27

**Authors:** Martina Lundqvist, Lars-Åke Levin, Kerstin Roback, Jenny Alwin

**Affiliations:** Department of Medical and Health Sciences, Linkoping University, Linköping, Sweden

**Keywords:** Assistance dog, Service dog, Alert dog, Health-related quality of life, Well-being, Self-esteem

## Abstract

**Background:**

Individuals with severe disability often require personal assistance and help from informal caregivers, in addition to conventional health care. The utilization of assistance dogs may decrease the need for health and social care and increase the independence of these individuals. Service and hearing dogs are trained to assist specific individuals and can be specialized to meet individual needs. The aim of this study was to describe and explore potential consequences for health-related quality of life, well-being and activity level, of having a certified service or hearing dog.

**Methods:**

A longitudinal interventional study with a pre-post design was conducted. At inclusion, all participants in the study had a regular (untrained) companion dog. Data were collected before training of the dog started and three months after certification of the dog. Health-related quality of life was assessed with EQ-5D-3L, EQ-VAS and RAND-36. Well-being was measured with WHO-5 and self-esteem with the Rosenberg Self-Esteem Scale. In addition, questions were asked about physical activity and time spent away from home and on social activities. Subgroups were analyzed for physical service and diabetes alert dogs.

**Results:**

Fifty-five owner-and-dog pairs completed the study (30 physical service dogs, 20 diabetes alert dogs, 2 epilepsy alert dogs, and 3 hearing dogs). Initially, study participants reported low health-related quality of life compared with the general population. At follow-up, health-related quality of life measured with the EQ-VAS, well-being and level of physical activity had improved significantly. In the subgroup analysis, physical service dog owners had lower health-related quality of life than diabetes alert dog owners. The improvement from baseline to follow-up measured with EQ-5D statistically differed between the subgroups.

**Conclusions:**

The target population for service and hearing dogs has an overall low health-related quality of life. Our study indicates that having a certified service or hearing dog may have positive impact on health-related quality of life, well-being and activity level. Service and hearing dogs are a potentially important “wagging tail aid” for this vulnerable population, able to alleviate strain, increase independence, and decrease the risk of social isolation.

**Trial Registration:**

The trial was retrospectively registered in http://clinicaltrial.gov, NCT03270592. September, 2017.

**Electronic supplementary material:**

The online version of this article (10.1186/s12913-018-3014-0) contains supplementary material, which is available to authorized users.

## Background

Individuals with multiple illnesses often require conventional health care as well as personal assistance and help from family and friends. They often have needs resulting in a high demand for health and social care resources [1]. Disabilities may also result in an increased risk of social isolation and thereby restrictions to an individual’s desired lifestyle [2]. Therefore it is essential to find means and measures that can give both physical as well as psychological support to decrease these individuals’ needs for health and/or social care, and to increase their independence. The use of an assistance dog may help achieve this.

Included in the assistance dog concept are guide dogs, hearing dogs and service dogs. Further, service dogs can be divided into subgroups of physical service dogs, diabetes alert dogs and seizure alert dogs etc. [3]. Physical service dogs are dogs that are specially trained to assist individuals with disabilities. They are commonly trained to pick up dropped items, carry items, help individuals get dressed, and move wheelchairs. The dogs are also trained to attract other people’s attention in case of emergency or if the owner needs help. Service dogs can also be specifically trained to meet the owners’ specific needs. For individuals with diabetes or epilepsy, specially trained service dogs, called diabetes and seizure alert dogs, warn their owners of high or low blood sugar levels or of imminent epileptic seizures. For individuals with hearing impairment, hearing dogs are trained to assist by alerting their owners to sounds, such as a doorbell, smoke alarm or alarm clock [4, 5]. In Sweden, service and hearing dog training is carried out in three different ways: by the owner in collaboration with a certified instructor, by the owner alone, or by a certified instructor. This means it is possible to purchase an already certified service dog [6].

If owners of certified service or hearing dogs can become more independent as a result of the dog’s assistance, there is reason to believe that they may experience benefits influencing their perceived quality of life (QoL). This has also been suggested in earlier research regarding the impact of service dogs on health-related quality of life (HRQoL) [7–9]. Furthermore, previous research has shown that service dogs may help improve well-being, self-esteem and an individual’s psychosocial situation [10–12]. However, due to small samples and problems with controlling for confounders, results from previous research are associated with some uncertainty. Additionally, there are few studies conducted using generic instruments to measure the effects of certified service and hearing dogs. This motivates further research to describe the service and hearing dog owner population, and to further explore the potential consequences of having a certified dog, using different validated generic instruments that enable comparisons between service and hearing dog owners, specific disease groups, and the general population.

The aim of this study was to describe and explore the potential consequences for HRQoL, well-being and activity level, of having a certified service or hearing dog.

## Method

A longitudinal interventional study with a pre-post design was conducted to explore the potential consequences of having a certified service or hearing dog. The study was approved by the regional ethics vetting board at Linköping University (No: 157/09).

### Population

Participants in the study were made up of a self-selected sample of people who had a regular companion dog, and were recruited to the study between 2009 and 2013. The inclusion criteria were (1) ≥16 years old, (2) having a companion dog and (3) being in need of a service or hearing dog (i.e. having mobility impairment, diabetes, epilepsy or a hearing disorder). All participants gave written informed consent to participate in the study.

### Intervention

The participant brought his or her own companion dog to the Swedish Association of Service Dogs (SAF), with the intention of training it to become a certified service or hearing dog. Initially the dog was examined by a veterinarian. Thereafter, the owner and the dog performed a minor suitability test under the supervision of staff from SAF. The aim of the minor suitability test was primarily to determine the dog's appropriateness, and to assess whether the owner was able to carry out the training of the dog. When the minor test was approved, the dog and owner, as a team, had to pass a major suitability test. The major suitability test determined the dog's responsiveness and obedience. If the major suitability test was passed, the owner and dog started the training. The owner trained the dog in collaboration with a certified instructor who was appointed by SAF. Together they made up a training plan. When the dog and the owner had reached a sufficient level in the education process a certification test took place. The certification test included a number of tests to assess if the dog had all the skills required. After the certification, the dog received a yellow cape with a logo that identified the dog as a service or hearing dog. To keep the status as a certified dog, the owner and the dog had to pass annual certification maintenance tests.

### Data collection

**Fig. 1:**
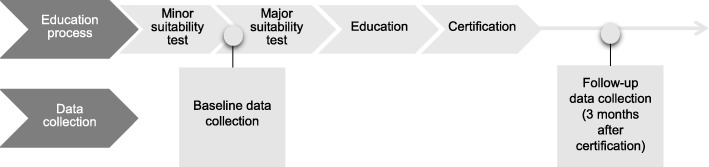
Education process and data collection procedure.

Baseline data were collected before the major suitability test or just before they started educating the dog (Fig. 1). Initially, the participants were contacted for a telephone interview. The telephone interview included questions regarding demographics, health care consumption, physical activity, and the dog’s assignments. After completion of the telephone interview, an additional questionnaire was sent by post to be completed by the participants. This questionnaire included self-assessment instruments (EQ-5D-3L, EQ-VAS, RAND-36, WHO-5, Rosenberg Self-Esteem Scale) and a number of open questions.

The follow-up data was collected three months after the owner and the dog completed the education and the dog was certified. The participants were once again contacted for a telephone interview and received an additional postal questionnaire. At the follow-up the same data was collected as at baseline.

### Instruments and questionnaires

In order to describe the population and explore the potential consequences of a certified service dog, several generic validated instruments were used.

#### EQ-5D

This HRQoL instrument includes the EQ-5D-3L descriptive system and the EQ-VAS [13]. The EQ-5D-3L comprises five dimensions: mobility, self-care, usual activities, pain/discomfort, and anxiety/depression. For each dimension respondents are asked to report their status on a three-level ordinal scale: “no problems”, “some problems”, or “severe problems”. The EQ-5D-3L health states can be combined into a single index, using a valuation formula based on valuations from population samples [13]. A score of 1 represents the HRQoL corresponding to perfect health, and 0 represents the HRQoL corresponding to death. The EQ-VAS is a standard vertical 20 cm visual analogue scale for recording an individual’s rating for their current HRQoL state.

#### RAND-36

This is an HRQoL instrument that includes 36 questions. There are thirty-five questions across eight dimensions: physical functioning (PF), physical role functioning (RP), bodily pain (BP), general health (GH), vitality (VT), social functioning (SF), emotional role functioning (RE), and mental health (MH). Each dimension is transformed to a scale from 0 (worst imaginable health state) to 100 (best imaginable health state). The questionnaire also includes a single item that provides an indication of perceived change in health: Health transition scale (HT) [14]. RAND-36 is a very closely correlated (0.99) open-source complement to the MOS 36-Item Short-Form Health Survey (SF-36) [15]. To be able to provide a single index score out of RAND-36, the SF-6D was used, ranging between 0.291 and 1, where 1 represents perfect health. The SF-6D was computed using the algorithm provided by Brazier et al. [16].

#### WHO-5

The instrument measures subjective well-being and comprises five items: feeling cheerful and in good spirits, feeling calm and relaxed, feeling active and vigorous, feeling fresh and rested, and meaningful daily life. The items are scored from 5 (all of the time) to 0 (none of the time). The raw score is multiplied by 4 to translate it to a percentage scale from 0 (absence of well-being) to 100 (maximal well-being) [17].

#### Rosenberg Self-Esteem Scale

The self-esteem questionnaire includes ten statements where each statement has a four-point Likert scale – from “Strongly agree” to “Strongly disagree”. The scale ranges from 0-30, with 30 indicating the highest score possible [18]. No Swedish version of the questionnaire was available. Therefore, a translation back-translation procedure was employed.

#### Other questions

A set of questions concerning physical activity, time spent outside the home and time spent on social activities was posed. To assess physical activity a question from the Lifestyle Report 2008 constructed by The Swedish National Institute of Public Health (SNIPH) was used [19]. The owners were asked to rate how much they had moved around and exerted themselves physically in their leisure time during the last 3 months, on a 4-point scale ranging from, “sedentary leisure time” to “regular exercise and training”. Sedentary leisure time meant that they spent most of their time on reading, television, cinema or other sedentary activities and moved around less than 2 hours a week. Regular exercise and training meant that they ran, swam, played tennis, played badminton, did gymnastics or similar at least 3 times per week, where each session lasted at least 30 minutes.

In addition, at the follow-up the participants were asked if the time they spent outside their home and the time they spent on social activities had changed (decreased, stayed the same or increased), since the dog became certified. The questions were developed by the research group for this study and were only asked at the follow-up interview.

### Statistical analyses

To determine the sample size, a power calculation was carried out based on the minimal important difference (MID) in the SF-6D. Using a Type I error rate of α=0.05, statistical power of 0.80, the MID of 0.041 for the SF-6D [20], and with an assumed standard deviation of the change of 0.01, a sample size of 47 participants was considered as a minimum.

In order to explore the potential consequences of a certified service dog, paired sample t-tests were applied. To determine the magnitude of the effect, Cohen's d was calculated. The effect sizes should be interpreted as small (d=0.2-0.5), moderate (d=0.5-0.8) or large (d>0.8) [21].

In addition to the pre-post comparisons, we also wanted to describe the population. The results from the EQ-5D-3L were therefore compared with the HRQoL values estimated with EQ-5D-3L for the general population [22], and the population norms for SF-6D [23]. In addition, the RAND-36 results were compared with Swedish general population estimates based on SF-36 [15].

Two subgroup analyses were also conducted. Since the dog owners make up a heterogeneous group, the participants were divided into two more homogeneous groups depending on type of dog: either (1) a physical service dog or (2) a diabetes alert dog. Independent t-tests were performed in order to test whether there was a difference between the subgroups. We performed all of the analyses with the statistics software package SPSS version 23.0 [24].

## Results

Sixty-nine owners and their dogs were enrolled in the study. Fifty-five of them became certified, and thus constitute our study population, see Additional file 1. The education process (or training) took on average 1.5 years.

The telephone interview was conducted on average 18 days (SD: 28.0 days) before the major suitability test. Four participants gave their baseline interview retrospectively, since the education of the dog had begun when the study enrollment started. The follow-up interview was conducted on average 95 days (SD: 22.7 days) after the certification of the dog.

### Baseline characteristics

Table 1 provides a summary of self-reported baseline characteristics from the 55 participants that completed the study. The average age was 44 years (range 17-68 years) when entering the study. The majority were on disability pensions or was employed part-time, and 40% lived in single households. The most common diseases/functional impairments that the participants cited as the reasons for needing a certified service dog were diabetes, neurological disorders, and musculoskeletal disorders.

**Table 1:** Baseline characteristics of the participants

Baseline characteristics, participants	Total (*n*=55)
Age	
Mean (SD)	43.8 (14.0)
Min – Max	17 – 68
Gender	
Female	47 (85.5%)
Education	
Primary school	6 (10.9%)
Medium level	15 (27.3%)
University degree	22 (40.0%)
Other	12 (21.8%)
Main activity/Professional status	
Employed full-time	5 (9.1%)
Employed part-time	13 (23.6%)
Student	4 (7.3%)
Sick leave	4 (7.3%)
Retired	2 (3.6%)
Disability pension	23 (41.8%)
Other	4 (7.3%)
Household arrangement	
A couple or more	33 (60.0%)
Single	22 (40.0%)
Disease/Functional impairment	
Diabetes	20 (36.4%)
Neurological disorder	15 (27.3%)
Musculoskeletal disorder	12 (21.8%)
Deaf/Hard of hearing	3 (5.5%)
Epilepsy	2 (3.6%)
Other	3 (5.4%)

Baseline characteristics of the dogs in the study are presented in Table 2. The mean age of the 55 dogs that completed the study was at baseline 2.2 years (range 1-4 years). Thirty dogs were trained as physical service dogs, 20 as diabetes alert dogs, 2 as seizure alert dogs, and 3 as hearing dogs (Table 2).

**Table 2:** Baseline characteristics of the dogs

Baseline characteristics, dogs	Total (*n*=55)
Age	
Mean (SD)	2.2 (0.7)
Min – Max	1.3 – 4.0
Weight (kg)	
Mean (SD)	22.6 (10.7)
Min-Max	3.2 – 52.0
Height (cm)	
Mean (SD)	49.8 (11.7)
Min-Max	19.0 – 67.5
Gender	
Bitch	27 (49.1%)
Neutered	
Yes	16 (29.1%)
No	39 (70.9%)
Assistance dog	
Physical service dog	30 (54.5%)
Diabetes alert dog	20 (36.4%)
Seizure alert dog	2 (3.6%)
Hearing dog	3 (5.5%)
Breed categories^a^	
Retrievers - Flushing Dogs - Water Dogs	21 (38.2%)
Sheepdogs and Cattle Dogs	12 (21.8%)
Companion and Toy Dogs	9 (16.4%)
Pinscher and Schnauzer - Molossoid Breeds - Swiss Mountain and Cattle Dogs	4 (7.3%)
Terriers	4 (7.3%)
Sighthounds	2 (3.6%)
Crossbreed	2 (3.6%)
Spitz and Primitive types	1 (1.8%)

^a^According to Federation Cynologique Internationale (FCI) [27]

The two most common breed categories in the study were "Retrievers – Flushing Dog – Water Dogs" and "Sheepdogs and Cattle dogs" (Table 2).

### Health-related quality of life

At baseline, the total study population mean reported EQ-5D index value was 0.441, the EQ-VAS value was 55.15, and the SF-6D value 0.639, Table 3. This is considerably lower than the general population with a mean EQ-5D index of 0.86, EQ-VAS of 87, and SF-6D of 0.79 [22, 23]. At the follow-up, the participants reported a statistically significant improvement of their HRQoL, measured with the EQ-VAS. The EQ-5D and SF-6D index scores also indicated an improvement, but the results were not statistically significant. Testing the magnitude of the effects with Cohen's d showed that the effects were small.

**Table 3:** HRQoL measures for the general population in Sweden and HRQoL measures for the total study population at baseline and follow-up

Instrument	n	General population^a^ (40-49 years)	Baseline (SD)	Follow-up (SD)	Diff.	p-value	Cohne's d^b^
The total study population							
EQ-5D single index	53	0.86	0.441 (0.363)	0.491 (0.339)	0.050	0.234	0.162
EQ-VAS	53	87	55.15 (21.125)	62.62 (19.450)	7.472	0.007^*^	0.384
SF-6D	52	0.79	0.639 (0.126)	0.650 (0.126)	0.011	0.441	0.111

^*^Statistically significant at a p-value level of 0.05 ^a^General population: index *n*=588 [22], VAS *n*=556 [22], SF-6D *n*=22 166 [23]. ^b^Cohen's d values: Small=0.2-0.5; Medium=0.5-0.8; Large>0.8

Table 4 shows the frequency of participants with a change in EQ-5D dimension scores between baseline and the follow-up. In the EQ-5D dimension scores “usual activities” and “anxiety/depression”, nearly a quarter of the participants improved. However, the majority remained unchanged.

**Table 4:** Change in EQ-5D dimension scores between baseline and follow-up

EQ-5D Dimensions	Change
Mobility (*n*=54)	
Improved	6 (11.1%)
No change	46 (85.2%)
Worsened	2 (3.7%)
Self-care (*n*=54)	
Improved	4 (7.4%)
No change	42 (77.8%)
Worsened	8 (14.9%)
Usual activities (*n*=54)	
Improved	13 (24.1%)
No change	36 (66.7%)
Worsened	5 (9.3%)
Pain/discomfort (*n*=53)	
Improved	6 (11.3%)
No change	41 (77.4%)
Worsened	6 (11.3%)
Anxiety/depression (*n*=54)	
Improved	13 (24.1%)
No change	35 (64.8%)
Worsened	6 (11.1%)

In comparison to the Swedish general population estimated SF-36 domain scores, all the RAND-36 domain scores for the participants were strikingly low [15]. There was a statistically significant improvement between baseline and follow-up in two of the eight RAND-36 domains: Physical Role functioning (RP) and Emotional Role functioning (RE) scores (Table 5), as well as for the summary score Health Transition (HT). In addition, there was a weak trend towards improvement in both Vitality (VT) and Mental Health (MH). However, the magnitude of the effects on the RAND-36 dimensions, calculated with Cohen's d, were small.

**Table 5:** Mean SF-36 scores (SD) estimates for the general population in Sweden and mean RAND-36 scores for the total study population at baseline and follow-up

HRQoL score (n=54)	SF-36 General population^a^ (SD) [15]	Baseline (SD)	Follow-up (SD)	Diff.	p-value	Cohen's d^b^
PF	87.9 (19.6)	47.8 (32.6)	45.5 (35.1)	-2.31	0.329	-0.134
RP	83.2 (31.8)	34.3 (40.1)	50.6 (40.7)	16.36	0.003^*^	0.423
BP	74.8 (26.1)	55.3 (31.2)	56.3 (30.4)	1.02	0.728	0.048
GH	75.8 (22.2)	46.0 (24.2)	43.1 (22.8)	-2.99	0.212	-0.172
VT	68.8 (22.8)	41.0 (25.0)	46.2 (23.5)	5.28	0.051	0.272
SF	88.6 (20.3)	63.4 (27.3)	66.7 (23.8)	3.30	0.352	0.129
RE	85.7 (29.2)	59.3 (41.3)	75.3 (40.0)	16.05	0.013^*^	0.348
MH	80.9 (18.9)	67.0 (20.2)	71.8 (19.8)	4.83	0.057	0.265
HT		42.1 (25.2)	52.3 (25.4)	10.19	0.020^*^	0.325

*PF* Physical Function, *RP* Role Physical, *BP* Bodily Pain, *GH* General Health, *VT* Vitality, *SF* Social Function, *RE* Role Emotional, *MH* Mental Health, *HT* Health Transition score. ^*^Statistically significant at a p-value level of 0.05. ^a^n=8930. ^b^Cohen's d values: Small=0.2-0.5; Medium=0.5-0.8; Large>0.8

### Well-being and self-esteem

The mean WHO-5 score for the participants at baseline was 48.4. At follow-up they had significantly improved to 54.7 (p-value: 0.030). There was also an indication of improvement in self-esteem between baseline and follow-up (p-value: 0.068).

### Physical activity and social functioning

**Fig. 2:**
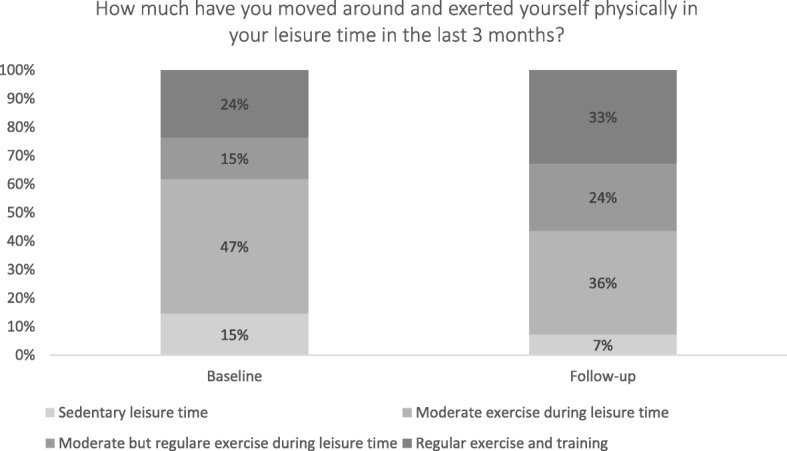
Distribution of physical activity during leisure time in the last 3 months

At the follow-up, a larger number of the participants stated that they regularly exercised and trained during their leisure time (33%), compared to baseline (24%), Fig. 2. The improvement was statistically significant (p-value: 0.021).

At the follow-up, 67 percent of the participants also stated that the proportion of time spent outside their home had increased, and 61 percent that they spent more time on social activities.

### Subgroup analysis

In the subgroup of owners of physical service dogs (excluding owners of alert or hearing dogs), the HRQoL measured with EQ-5D single index, EQ-VAS and SF-6D, was very low. In comparison, owners of diabetes alert dogs reported higher HRQoL (Table 6). Both groups tended to improve at the follow-up, but the improvements were not statistically significant. However, the improvement from baseline to follow-up measured with EQ-5D statistically differed between the subgroups (p-value: 0.045).

**Table 6:** HRQoL measures for the general population in Sweden and HRQoL measures for physical service dog owners and diabetes alert dog owners at baseline and follow-up

Instrument	n	General population^a^ (40-49 years)	Baseline (SD)	Follow-up (SD)	Diff.	p-value	Cohen's d^b^
Physical service dogs							
EQ-5D single index	29	0.86	0.266 (0.323)	0.351 (0.282)	0.086	0.201	0.243
EQ-VAS	29	87	52.97 (22.301)	60.93 (17.625)	7.966	0.058	0.367
SF-6D	28	0.79	0.590 (0.093)	0.610 (0.088)	0.020	0.282	0.208
Diabetes alert dog							
EQ-5D single index	19	0.86	0.656 (0.277)	0.674 (0.336)	0.017	0.741	0.077
EQ-VAS	19	87	56.00 (20.685)	63.89 (23.120)	7.895	0.075	0.434
SF-6D	19	0.79	0.699 (0.143)	0.719 (0.143)	0.020	0.424	0.188

^a^General population: index *n*=588 [22], VAS *n*=556 [22], SF-6D *n*=22 166 [23] ^b^Cohen’s d values: Small=0.2-0.5; Medium=0.5-0.8; Large>0.8

In comparison with the Swedish general population, owners of physical service dogs also had very low RAND-36 domain scores, see Additional file 2. However, a statistically significant improvement was found in the following RAND-36 domains: Physical Role functioning (RP), Emotional Role functioning (RE), and the Health Transition score (HT). The Cohen's d values indicated small effects in all domains scores except in the RP domain, where the effect was determined as medium [Additional file 2]. No differences were found in RAND-36 between baseline and follow-up for owners of diabetes alert dogs, [Additional file 3]. In addition, no statistically significant differences were found between the subgroups.

## Discussion

For individuals suffering from multiple illnesses, it is necessary to find assistant strategies that can support both physical and psychological needs. In this study we have explored the potential consequences for HRQoL, well-being and activity level, of having a certified service or hearing dog, using a pre-post study approach. Overall, the results indicate that there may be positive consequences of having a certified service dog in terms of HRQoL, well-being and activity level. Specifically, statistically significant improvements found in RAND-36 indicate that the participants in the study experienced a potential reduction in their mental difficulties based on their tasks and daily activities, and a change in general health during the past year. Further, well-being and the degree of physical activity during leisure time had improved statistically at the follow-up. At the follow-up the participants also declared that time spent outside their home and time spent on social activities had increased, which was in line with the improvements in RAND-36. However, using Cohen's d to express the magnitude of the difference between baseline and follow-up, showed that the effects in general were small.

When comparing the HRQoL scores in this study with HRQoL data for the general population measured with EQ-5D, it may be noted that HRQoL scores for the general population are considerably higher than for the participants in our study. The EQ-5D mean value for people of 40-49 years of age in Sweden is 0.86 [22], and in the present study 0.44-0.49. This is confirmed by comparing results from the study using RAND-36 with data for the general population measured with SF-36. Participants in our study have lower scores in all eight domains. Overall, the comparisons show that the participants in our study have a remarkably poor HRQoL, and can be considered, as a population, to be in bad condition and to have substantial needs.

When dividing the total study population into subgroups, we found that physical service dog owners had low HRQoL. The subgroup analysis for owners of a diabetes alert dog showed in comparison higher HRQoL scores. However, the change between baseline and follow-up measured with EQ-5D statistically differed between the groups, indicating that physical service dog owners benefited more of a certified dog, in terms of HRQoL.

Previous studies measuring assistance dog impact on HRQoL have shown various results. Shintani et al. reported improvements for service dog owners (*n*=10) in the subscales Role Function (RE), Physical Function (PF), and in the Mental Component Summary measured with the SF-36 v2 instrument, compared to the control group (*n*=28) [8]. Hubert et al. measured the service dog impact on QoL, in individuals using manual wheelchairs. They found no significant change in perceived QoL measured with Quality of Life Index (QLI) at baseline, compared to the follow-up for individuals receiving a service dog (*n*=13) [7]. Previous studies measuring self-esteem of service dog owners have also shown various results. Collins et al. found no significant differences between the service dog group and the comparison group [12]. Allen et al., studying individuals with severe ambulatory disabilities, found that individuals receiving a service dog had significantly improved self-esteem [11]. In our study the participants’ self-esteem also improved, but the result was not statistically significant. In a study conducted by Fairman et al., service dog owners reported that they had increased their social interactions, and that they felt that it was easier to leave their home since they had acquired a service dog [25]. Camp et al. also found that service dog owners had increased their social contacts and their participation in activities since acquiring a service dog [10]. In our study the majority of the participants reported at the follow-up that they had increased the time they spend outside their home, and the time they spent on social activities. This may have influenced the HRQoL improvement. According to White et al., attachment style is related to quality of life for assistance dog owners [26]. It is possible that this has had an impact on our results as well, but this has not been studied.

To our knowledge, this study is the largest study to explore the potential consequences for HRQoL, well-being and activity level, of having a certified service or hearing dog. Another strength is that we used several validated generic instruments to assess HRQoL. Further, there are only a few missing values for the participants that completed the study. However, as in most comparable studies, it was not possible to include a randomized control group in this study, since we, for ethical reasons, were unable to exclude a group of participants from the service dog education. The lack of a control group creates challenges and may have affected the results by a Hawthorne effect. Another limitation of the pre-post design is the lack of control for confounders. For example, it was not possible to control for disease progressiveness, although many of the participants in our study had progressive diseases, which may have led to an underestimation of the results.

As stated before, the aim of this study was to explore the potential consequences of having a certified service or hearing dog. However, the study design was not optimal. By controlling for confounders, e.g. conducting a randomized controlled trial, we have reason to believe that a future improved attempt to evaluate service and hearing dogs could imply more favorable effects of certified service and hearing dogs. Furthermore, since we only had a three-month follow-up in the present study, long-term effects have not been captured. There is reason to believe that the skills of the dog, and the collaboration and attachment between owner and dog will develop over time. It is therefore of importance to conduct additional research that makes it possible to take into account the long-term effects of a certified service or hearing dog, both in terms of costs, and health outcomes evaluating the cost-effectiveness. Interestingly though, the present study indicates that educating a regular companion dog to become a certified service or hearing dog may improve health outcomes for a complex patient group with substantial needs. In addition, a certified service or hearing dog might be a competing alternative that offers a behavior that humans interpret as happy, friendly and devoted.

## Conclusions

The study reveals that the target population for service or hearing dogs has a low HRQoL. Furthermore, the study indicates that there may be positive consequences of having a certified service and hearing dog in terms of HRQoL, well-being and activity level. When assistance dogs are able to alleviate strain, increase an individual’s independence, decrease the risk of social isolation and improve well-being, they are a potentially important aid with wagging tails, suitable for a vulnerable population of this kind.

## Additional files

Additional file 1: Flow chart of the work process. A modified version of the CONSORT 2010 Flow Diagram. (DOC 28 kb)

Additional file 2: RAND-36 scores for owners of a physical service dog. Mean RAND-36 scores for owners of a physical service dog at baseline and follow-up. (DOCX 17 kb)

Additional file 3: RAND-36 scores for owners of a diabetes alert dog. Mean RAND-36 scores for owners of a diabetes alert dog at baseline and follow-up. (DOCX 17 kb)

## Abbreviations

BP: Bodily Pain
GH: General Health
HRQoL: Health-Related Quality of Life
HT: Health Transition score
MH: Mental Health
PF: Physical Function
QLI: Quality of Life Index
QoL: Quality of Life
RE: Role Emotional
RP: Role Physical
SD: Standard Deviation
SF: Social Function
VT: Vitality
