# Supplementary material for: The impact of service and hearing dogs on health-related quality of life and activity level: a Swedish longitudinal intervention study
Source: BMC Health Serv Res. 2018 Jun 27;18:497. doi: 10.1186/s12913-018-3014-0 (PMC6020368; doi:10.1186/s12913-018-3014-0)
Supplement: Supplementary file 1 — Flow chart of the work process. A modified version of the CONSORT 2010 Flow Diagram. (DOC 28 kb) [file 12913_2018_3014_MOESM1_ESM.doc]

**Flow chart of the work process**

**Baseline**

**Analysis**

**Follow-Up**

**Enrollment**

Enrolled in the study: passed the minor suitability test (n=69)

Drop outs (n=14)
♦Personal reasons n=6
♦The dog was not able to complete education n=3
♦Participant did not complete the education n=2
♦Unknown reason n=2
♦Illness n=1

Baseline data collected (n=69)

Follow-up data collected: study population (n=55)

Analysed (n=55)
