# Supplementary material for: The impact of service and hearing dogs on health-related quality of life and activity level: a Swedish longitudinal intervention study
Source: BMC Health Serv Res. 2018 Jun 27;18:497. doi: 10.1186/s12913-018-3014-0 (PMC6020368; doi:10.1186/s12913-018-3014-0)
Supplement: Supplementary file 2 — RAND-36 scores for owners of a physical service dog. Mean RAND-36 scores for owners of a physical service dog at baseline and follow-up. (DOCX 17 kb) [file 12913_2018_3014_MOESM2_ESM.docx]

# **RAND-36 scores for owners of a physical service dog**

| **HRQoL score (n=30)** | **SF-36 General population† (SD) [15]** | **Baseline (SD)** | **Follow-up (SD)** | **Diff.** | **p-value** | **Cohen's d‡** |
| --- | --- | --- | --- | --- | --- | --- |
| **PF** | 87.9 (19.6) | 26.8 (22.2) | 24.3 (22.6) | -2.50 | 0.205 | -0.237 |
| **RP** | 83.2 (31.8) | 23.3 (34.1) | 44.4 (36.8) | 21.11 | 0.007* | 0.534 |
| **BP** | 74.8 (26.1) | 44.8 (30.7) | 48.5 (27.4) | 3.75 | 0.321 | 0.184 |
| **GH** | 75.8 (22.2) | 46.4 (22.4) | 44.0 (20.8) | -2.39 | 0.462 | -0.136 |
| **VT** | 68.8 (22.8) | 40.2 (23.4) | 47.0 (19.3) | 6.83 | 0.053 | 0.368 |
| **SF** | 88.6 (20.3) | 55.6 (26.4) | 63.8 (23.2) | 8.19 | 0.079 | 0.339 |
| **RE** | 85.7 (29.2) | 56.7 (43.9) | 77.8 (37.5) | 21.11 | 0.019* | 0.452 |
| **MH** | 80.9 (18.9) | 66.7 (19.1) | 72.3 (18.7) | 5.63 | 0.090 | 0.320 |
| **HT** |  | 36.7 (25.2) | 50.0 (27.1) | 13.33 | 0.040* | 0.393 |
| PF=Physical Function; RP=Role Physical; BP=Bodily Pain; GH=General Health; VT=Vitality; SF=Social Function; RE=Role Emotional; MH=Mental Health; HT=Health Transition score. *Statistically significant at a p-value level of 0.05 †n=8930. ‡Cohen's d values: Small=0.2-0.5; Medium=0.5-0.8; Large>0.8 | | | | | | |
