# Supplementary material for: The impact of service and hearing dogs on health-related quality of life and activity level: a Swedish longitudinal intervention study
Source: BMC Health Serv Res. 2018 Jun 27;18:497. doi: 10.1186/s12913-018-3014-0 (PMC6020368; doi:10.1186/s12913-018-3014-0)
Supplement: Supplementary file 3 — RAND-36 scores for owners of a diabetes alert dog. Mean RAND-36 scores for owners of a diabetes alert dog at baseline and follow-up. (DOCX 17 kb) [file 12913_2018_3014_MOESM3_ESM.docx]

# **RAND-36 scores for owners of a diabetes alert dog**

| **HRQoL score (n=19)** | **SF-36 General population† (SD) [15]** | **Baseline (SD)** | **Follow-up (SD)** | **Diff.** | **p-value** | **Cohen's d‡** |
| --- | --- | --- | --- | --- | --- | --- |
| **PF** | 87.9 (19.6) | 75.8 (21.6) | 80.3 (19.3) | 4.47 | 0.154 | 0.341 |
| **RP** | 83.2 (31.8) | 44.7 (41.3) | 59.2 (44.3) | 14.47 | 0.142 | 0.352 |
| **BP** | 74.8 (26.1) | 68.7 (26.7) | 68.3 (29.4) | -0.39 | 0.932 | -0.020 |
| **GH** | 75.8 (22.2) | 41.6 (28.4) | 39.2 (25.7) | -2.37 | 0.579 | -0.130 |
| **VT** | 68.8 (22.8) | 41.1 (29.2) | 46.3 (27.4) | 5.26 | 0.283 | 0.254 |
| **SF** | 88.6 (20.3) | 71.7 (24.9) | 76.3 (21.2) | 4.61 | 0.359 | 0.216 |
| **RE** | 85.7 (29.2) | 61.4 (37.3) | 78.9 (37.2) | 17.54 | 0.056 | 0.468 |
| **MH** | 80.9 (18.9) | 66.7 (22.9) | 73.9 (18.5) | 7.16 | 0.083 | 0.421 |
| **HT** |  | 46.1 (25.4) | 57.9 (18.7) | 11.84 | 0.058 | 0.464 |
| PF=Physical Function; RP=Role Physical; BP=Bodily Pain; GH=General Health; VT=Vitality; SF=Social Function; RE=Role Emotional; MH=Mental Health; HT=Health Transition score. †n=8930. ‡Cohen's d values: Small=0.2-0.5; Medium=0.5-0.8; Large>0.8 | | | | | | |
